# Digital Acoustic Uroflowmetry for Noninvasive Urine Flow Rate Monitoring in Men Using Smartphone Acoustic Pattern Recognition: Protocol for the Development of a System and Mobile App

**DOI:** 10.2196/102842

**Published:** 2026-07-23

**Authors:** Kevin Yonathan, Harrina Erlianti Rahardjo, Irfan Wahyudi, Fina Widia, Putu Angga Risky Raharja, Saras Serani Sesari

**Affiliations:** 1 Division of Urology, Department of Uro-Nephrology Faculty of Medicine University of Indonesia Jakarta, DKI Jakarta Indonesia; 2 Department of Urology University of Indonesia Hospital Depok, West Java Indonesia

**Keywords:** acoustic uroflowmetry, digital recognition, lower urinary tract symptoms, mobile health, uroflowmetry

## Abstract

**Background:**

Lower urinary tract symptoms (LUTS) constitute a significant global health burden with a severe impact. Uroflowmetry, the gold standard assessment for measuring urinary flow, may be inaccessible due to equipment availability or cost. Meanwhile, the proliferation of smartphones, even in resource-limited countries, offers a promising infrastructure for developing an innovative tool. Using the built-in microphone of smartphones to capture and analyze voiding sounds to estimate urine flow parameters has emerged as a potential solution to overcome these limitations.

**Objective:**

This study aims to develop a digital acoustic uroflowmetry system and a mobile app based on acoustic pattern recognition for the noninvasive estimation of key urine flow parameters.

**Methods:**

This protocol describes a staged observational study design comprising four distinct phases: (1) app development, (2) algorithm training and model optimization, (3) internal validation, and (4) independent clinical testing and comparison with conventional uroflowmetry. A smartphone app will be developed to record voiding sounds. Customized signal processing algorithms will be designed to analyze acoustic signals and estimate urine flow parameters based on these signals. Participants will be recruited from urology clinics, and each participant will undergo measurement using both conventional uroflowmetry and the acoustic uroflowmetry app. The app will guide users on proper smartphone placement during voiding to ensure input quality. Acoustic features will be extracted, and models will be trained and validated. The primary outcome will be the correlation and agreement between the values of maximum flow rate (Q_max_), average flow rate (Q_avg_), and voided volume (VV) measured by the acoustic uroflowmetry app and those measured by conventional uroflowmetry.

**Results:**

This study is currently at the protocol development stage and received funding in May 2026. The first phase (development of the system and mobile app) started in December 2025, and the recruitment of participants for the first phase is planned to start in July 2026. The study results are expected to be available by early 2027.

**Conclusions:**

The development of a digital acoustic uroflowmetry system using acoustic pattern recognition is intended to enhance the accessibility and convenience of urine flow monitoring, particularly for patients in regions with limited health care infrastructure.

**International Registered Report Identifier (IRRID):**

PRR1-10.2196/102842

## Introduction

Lower urinary tract symptoms (LUTS) encompass a spectrum of bothersome conditions affecting a substantial proportion of the global population, significantly diminishing quality of life and imposing a considerable economic burden on health care systems worldwide [1]. An estimated 2.3 billion people globally are affected by LUTS [1]. The assessment of urinary flow dynamics (uroflowmetry) is the gold standard noninvasive method used in the diagnostic evaluation of these conditions. It provides critical parameters, such as maximum flow rate (Q_max_), average flow rate (Q_avg_), voided volume (VV), and flow time (FT), which are essential for forming diagnoses, guiding treatment decisions, and monitoring therapeutic outcomes [2]. Traditionally, uroflowmetry is performed in specialized clinical settings using dedicated equipment. Although it is considered the clinical gold standard, it has several inherent limitations. Access to uroflowmetry can be constrained by geographic location, especially in underdeveloped regions, as well as by the availability of specialized equipment and trained personnel. Furthermore, the need to travel to a suitable clinic for testing can be inconvenient and time consuming, leading to delays in diagnosis and suboptimal monitoring [3]. These barriers are particularly pronounced in resource-limited countries with uneven health care infrastructures.

The rapid adoption of smartphones, which are equipped with increasingly sophisticated sensors and powerful processors, has opened new frontiers in health care delivery, potentially improving access to various health care infrastructures. Even in resource-limited countries, the ownership and use of smartphones continue to rise. For example, data from Indonesia in 2024 indicated that 68.65% of the population owned mobile phones [4]. Previous studies have found that acoustic uroflowmetry is an alternative to conventional uroflowmetry that leverages the built-in microphone of a smartphone to record the acoustic signals generated during urination. By analyzing specific characteristics of these voiding sounds, such as intensity, frequency spectrum, and temporal patterns, sophisticated algorithms can be developed to estimate urine flow parameters [5-7]. The fundamental premise is that the acoustic signature produced when the urine stream impacts the toilet water surface correlates with urine flow rate. This approach offers a compelling solution to the limitations of conventional uroflowmetry. It requires no additional hardware beyond the smartphone itself, is highly portable, and enables patients to perform tests in the privacy and convenience of their own homes [5]. This creates a potential for home-based monitoring, enabling more frequent assessments, early detection of changes in urinary function, and timely intervention. Moreover, it may also serve as an alternative to traditional bladder diaries by providing a more precise and convenient approach for patients [5].

Despite these promising developments, the path to widespread clinical adoption of smartphone-based acoustic uroflowmetry apps is fraught with challenges that must be systematically addressed. The acoustic characteristics of voiding sounds are inherently variable and can be influenced by a multitude of factors, including the specifics of smartphones, the distance and orientation of the phone relative to the sound source, the shape and size of the toilet bowl, the water level, ambient environmental noise, and even individual variations in urinary stream characteristics [1,4,8]. These variabilities pose significant demands on the generalizability of the app’s underlying algorithms. Accurately translating complex acoustic signals into reliable and clinically meaningful urine flow parameters necessitates advanced signal processing techniques and robust model development [9,10]. Moreover, clinical validation must be used to demonstrate the accuracy, reliability, and consistency of the app when compared with the established gold standard [11].

This protocol is designed to provide a standardized framework for the development and validation of an acoustic uroflowmetry mobile app, using a clearly staged design that progresses from app development to algorithm training and model development, followed by clinical validation against conventional uroflowmetry.

## Methods

### Study Design

This study uses a staged observational cross-sectional design to develop and validate a digital acoustic uroflowmetry system using audio recordings captured by the built-in microphone of a smartphone. The study is organized into four sequential phases:

#### App Development and Acoustic Data Acquisition

In the first phase, the mobile app will be developed for the Android platform, with subsequent iOS development. The app will be designed to capture standardized acoustic recordings of voiding events using the built-in microphone. During acoustic data acquisition, simultaneous conventional uroflowmetry measurements will be obtained as the reference standard (paired recordings). The data obtained in this phase will be stored in a secure, encrypted database.

#### Algorithm Training and Model Optimization

The dataset collected from phase 1 will be randomly allocated into a training set (70%) and an internal validation set (30%). The training set will be used for feature extraction, model selection, and parameter tuning. Multiple candidate models using different approaches, such as convolutional neural networks (CNNs) and recurrent neural networks (RNNs), will be trained and compared.

#### Internal Validation

The best-performing model from phase 2 will undergo internal validation using the internal validation dataset (30%). Performance metrics, including correlation coefficients, intraclass correlation coefficients (ICCs), and Bland-Altman analyses, will be computed. If the performance fails to reach the clinically acceptable accuracy threshold, the model will be retrained with adjusted parameters before proceeding to the next phase.

#### Independent Clinical Testing and Comparison (External Validation)

A new independent sample of participants will be recruited for external validation. These participants will simultaneously undergo both acoustic uroflowmetry and conventional uroflowmetry measurements, and the finalized model will be applied without any further tuning.

A summary of the staged study design, including each phase, its objective, the corresponding dataset, and the expected output, can be found in Table 1.

**Table 1:** Summary of the staged study design.

Phases	Objectives	Datasets	Key outputs
1	App development and acoustic data acquisition	All enrolled participants	Mobile app Initial paired acoustic and conventional recordings database
2	Algorithm training and model optimization	Randomly selected 70% of the phase 1 dataset	Multiple trained candidate models Optimized model parameter
3	Internal validation	Remaining 30% of the phase 1 dataset	Internal performance validation metrics Final model selection
4	Independent clinical testing and comparison (external validation)	New participant sample (not included in phases 1-3)	Primary study outcomes

### Ethical Considerations

The study protocol was approved by the Medical Research Ethics Committee of Universitas Indonesia (KET-614/UN2.F1/ETIK/PPM.00.02/2026). The study will be conducted in accordance with the Declaration of Helsinki and its subsequent amendments, as well as with all applicable regulations.

Consent for the collection and publication of the data needed for this study will be obtained from participants as part of the standard of care, and the collected data will be used as a clinical reference. Informed consent will be obtained after the participants have received an explanation of the study from the researcher.

The data for this study will be collected by the participants using their own mobile phones. The data will then be securely stored in our encrypted database without any personal identifiers. The data will be accessible only to the participants and the researcher. The data chosen as examples for publication will also be deidentified according to the requirements of the relevant publication.

This protocol was prepared according to the Standards for Reporting Diagnostic Accuracy Studies for Artificial Intelligence (STARD-AI) guidelines [12]. Moreover, we used the Transparent Reporting of a Multivariable Prediction Model for Individual Prognosis or Diagnosis (TRIPOD) guidelines for model development in this study [13]. If the items from these guidelines overlap, the most detailed applicable recommendation will be adopted.

### Eligibility Criteria and Recruitment Procedures

The eligibility criteria for participants in this study are presented in Textbox 1.

**Inclusion, exclusion, and dropout criteria for study participation.**

**Inclusion criteria**

- Male sex and age ≥18 years
- Presence of lower urinary tract symptoms requiring conventional uroflowmetry as part of standard clinical assessment
- Ability to understand the study and provide informed consent
- Ability to operate the smartphone app independently or with minimal assistance
- Willingness to comply with the study procedures, including performing the acoustic uroflowmetry test as instructed

**Exclusion criteria**

- Presence of active urinary tract infection
- Inability to void spontaneously (eg, acute urinary retention or dependence on indwelling or intermittent catheterization for bladder emptying)
- History of recent urological surgery or instrumentation that may affect voiding parameters
- Presence of cognitive or hearing impairment that would interfere with understanding the study instructions or complying with the study procedures

**Dropout criteria**

- Withdrawal of consent at any stage of the study
- Inability to complete both conventional uroflowmetry and acoustic uroflowmetry tests as per the study protocol

This study is restricted to male participants for several reasons. First, the acoustic characteristics of voiding sounds differ fundamentally between men and women. Men typically void in a standing position, generating a urine stream that impacts the toilet water surface from a greater height and produces a distinct acoustic signature compared to women, who typically void in a seated position. The differences in acoustic signatures require separate algorithm development and validation [11]. A previous study showed that voiding acoustic signature measurements in female participants had a weak correlation with Q_max_ measurements [14].

The second reason is that LUTS in men, particularly LUTS secondary to benign prostatic hyperplasia (BPH), represent the most prevalent indication for uroflowmetry in daily clinical practice, and initial validation in this homogeneous population will reduce confounding variables during the model development phase [15,16]. Although LUTS are also prevalent among women, future validation will require separate algorithm development and validation.

Participants will be recruited consecutively. Eligible individuals will be provided with detailed information about the study using a standardized information sheet and will be given the opportunity to ask questions prior to enrollment. Those who agree to participate will be asked to sign an informed consent form.

### Clinical Outcomes: Uroflowmetry Parameters

The clinical outcomes measured in this study are described in Table 2. Before data collection begins, the clinically acceptable accuracy limits for each uroflowmetry parameter will be established. The limits will be established and adjusted in accordance with the published literature on uroflowmetry measurement variability and clinical decision-making thresholds [17]. The clinically acceptable accuracy limits for each parameter are presented in Table 2.

**Table 2:** Clinically acceptable accuracy limits for acoustic uroflowmetry parameters.

Parameter	Description	Target agreement metrics
Maximum flow rate (Q_max_)	The highest flow rate achieved during voiding, expressed in mL/s	ICC^a^ ≥0.85; LOA^b^ within +2 mL/s to –2 mL/s
Average flow rate (Q_avg_)	The mean flow rate over the entire voiding period, expressed in mL/s	ICC ≥0.80; LOA within +1.5 mL/s to –1.5 mL/s
Voided volume	The total volume of urine expelled during voiding, expressed in mL (the app may estimate this parameter based on flow rate and time)	ICC ≥0.80; LOA within +25% to –25%
Flow time	The total duration of urine flow, from the start to the end of voiding, expressed in seconds	ICC ≥0.80; LOA within +3 s to –3 s
Time to maximum flow (TQ_max_)	The elapsed time from the commencement of voiding to the point of Q_max_, expressed in seconds	ICC ≥0.75; LOA within +3 s to –3 s
Flow curve pattern	A qualitative assessment of the shape of the flow rate curve (eg, bell-shaped, plateau, or obstructive pattern; the app may also attempt to classify the flow curve pattern using machine learning techniques)	κ ≥0.70 (substantial agreement)

^a^ICC: intraclass correlation coefficient.

^b^LOA: limits of agreement.

### App Development, Training, and Testing

The acoustic uroflowmetry app will initially be developed for the Android operating system, followed by subsequent iOS development. The core components of the system are described in this section.

#### Acoustic Data Acquisition Module

**Figure 1:**
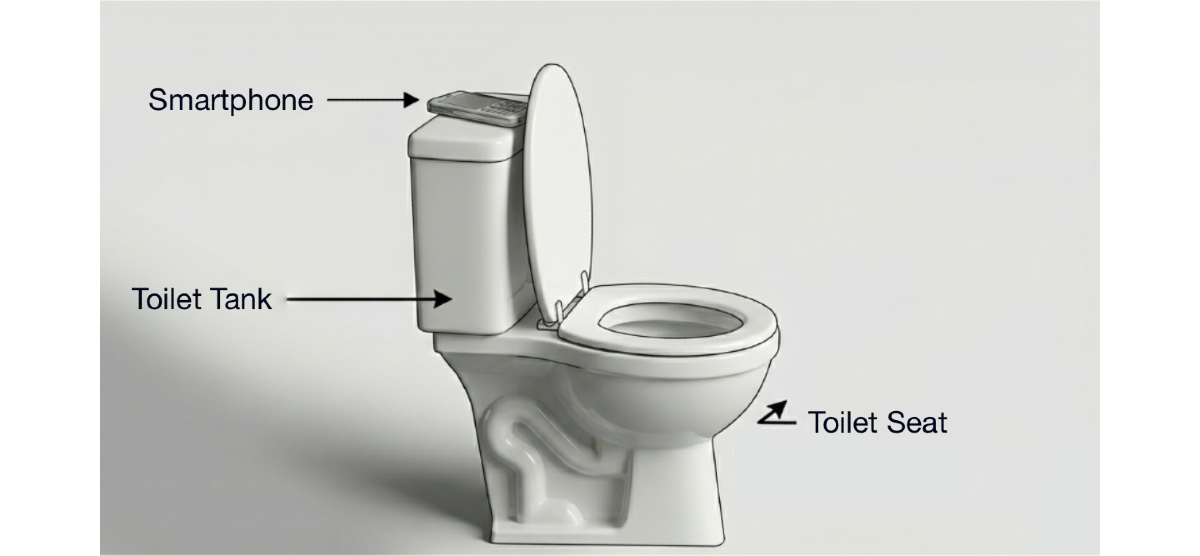
Example of smartphone positioning during data capture.

The app will use the smartphone’s built-in microphone. Audio recording parameters will be standardized (eg, a sampling rate of 44.1 kHz, 16-bit pulse-code modulation [PCM], and a mono channel). The app will incorporate clear, user-friendly visual and/or text-based instructions to guide users in optimal smartphone placement relative to the toilet bowl or to a standardized collection tool to ensure consistent audio capture (Figure 1).

#### Signal Preprocessing Module

Recorded audio will undergo preprocessing to enhance the quality of the voiding sound signal and mitigate environmental noise. Preprocessing may include filtering and baseline background noise calibration. Voiding onset and termination will be detected automatically within the audio stream based on relevant acoustic events.

#### Feature Extraction Module

A comprehensive set of acoustic features will be extracted from the segmented voiding sound signals. These may include time-domain features (eg, root mean square [RMS] energy, zero-crossing rate, and amplitude envelope), frequency-domain features (eg, spectral bandwidth and dominant frequency), and time-frequency features.

#### Flow Rate Estimation Module

The core of the app will be an algorithm that maps the extracted acoustic features to urine flow rate estimates using regression models or deep learning models, such as CNNs or RNNs.

#### Calibration Mechanism

The app will incorporate a calibration routine to account for interdevice variability in microphone sensitivity and background noise levels by recording a short ambient noise sample before voiding.

#### Reminder Module

The app will be able to provide daily reminders through push notifications to improve user compliance.

#### Model Testing

The dataset collected from phase 1 will be randomly partitioned into a training set (70% of recordings) and a hold-out internal validation set (30% of recordings) using random sampling. The performance of the trained models will be evaluated on a separate dataset. This dataset will not be used for model training or parameter tuning (Figure 1).

### Sample Size Estimation and Data Analysis

The sample size was estimated for a prespecified power of 90% and an α level of <.05. On the basis of prior studies comparing acoustic uroflowmetry with conventional uroflowmetry, the expected correlation coefficient (*r*) is approximately 0.8. Using the Fisher z transformation method to test the significance of the correlation coefficient against a null hypothesis of *r*=0.50 (the minimum correlation considered clinically meaningful), the required sample size is 38 paired observations. To account for an anticipated dropout rate of approximately 10%, the adjusted target sample size is 42 participants for phases 1 to 3 (70% allocated to phase 2 and 30% allocated to phase 3). Moreover, for the independent clinical testing phase (phase 4), a separate sample of 30 participants will be recruited based on Bland-Altman recommendations that a minimum of 30 paired measurements provides adequate precision for estimating the limits of agreement (LOA) [18]. Therefore, a total of 72 participants will be recruited for the study.

The clinical characteristics of the participants and uroflowmetry parameters will be presented descriptively. The analysis will be performed using SPSS for Macintosh (version 25.0; IBM Corp). The data will be deemed statistically significant if the *P* value is <.05.

Pearson correlation coefficient (*r*) will be used to assess the linear relationship between continuous uroflowmetry parameters (Q_max_, Q_avg_, VV, FT, and TQ_max_) obtained from the acoustic uroflowmetry app and conventional uroflowmetry. For data that do not meet the assumptions of normality, Spearman rank correlation coefficient (ρ) will be used instead.

The agreement between the 2 measurement methods will be evaluated using ICCs for absolute agreement. Bland-Altman plots will be constructed to visualize the bias (mean difference) and 95% LOA between the app and the gold standard measurements for Q_max_, Q_avg_, and VV. All agreement metrics will be compared with the predefined clinically acceptable accuracy limits outlined in Table 2. Moreover, qualitative classification of flow curves obtained using the app and those determined by an expert will be measured using Cohen κ.

## Results

The study was funded in May 2026 following peer review by the funder (Multimedia Appendix 1). The first phase (development of the system and mobile app) started in December 2025, and the recruitment of participants for the first phase is planned to start in July 2026. The study results are expected to be available by early 2027.

## Discussion

The development of a reliable acoustic uroflowmetry app has the potential to transform LUTS management by providing a readily accessible tool for urine flow monitoring. This protocol outlines a comprehensive framework for the development and clinical validation of such an app, addressing key aspects ranging from technical implementation to statistical analysis.

Although urodynamic assessment already has widespread use in developed countries, access to such assessments remains limited, particularly for individuals in remote or underdeveloped areas and those with mobility limitations. This limitation is addressed using the app by enabling testing in the home environment [1,3,4]. The decentralization of care could lead to earlier detection of urinary flow abnormalities, facilitate more timely interventions, and improve long-term outcomes for patients with lower urinary tract conditions such as BPH, urethral stricture, and neurogenic bladder [19]. The ability to perform frequent and convenient home-based monitoring could provide clinicians with more comprehensive datasets for assessment, moving beyond the limited snapshot provided by a single clinic-based measurement, which may be affected by factors such as insufficient urine volume or technical errors. Moreover, it could provide data similar to those obtained from a bladder diary with greater convenience by eliminating the need for manual urine collection during each micturition [20].

A primary concern associated with this innovation is ensuring the accuracy and reliability of acoustic-based flow estimations across a wide range of real-world conditions. Variability in smartphone hardware, environmental acoustics, and user technique can introduce significant noise and bias into the measurements [8,21-24]. Problems such as weak or absent signals and patient compliance need to be addressed. Our protocol addresses these challenges through attention to signal processing algorithms, baseline noise calibration, standardized user guidance for smartphone placement, reminder features, and the potential use of machine learning for pattern recognition. Moreover, clinical validation against the gold standard is a crucial step in establishing credibility and the potential clinical utility of the app.

Several pioneering studies have begun to explore the feasibility and validity of acoustic uroflowmetry, with variable results. One of the previous studies on this method was conducted by El Helou et al [1], who proposed and evaluated a novel mobile acoustic uroflowmetry method, demonstrating a strong correlation between flow rates estimated from smartphone-recorded sound signals and conventional uroflowmetry. Other studies, such as that by Dawidek et al [25], found that there was only a very low correlation for maximum flow measurement using audio-based uroflowmetry, thereby necessitating further research. Commercial ventures have also emerged, claiming to use AI to analyze urination sounds and provide flow metrics, signaling a growing interest in this technology within the consumer market [5]. Furthermore, comparative studies, such as those conducted by Song et al [7] and Rangganata et al [8], have evaluated mobile acoustic uroflowmetry apps against conventional uroflowmetry, further investigating their potential as reliable alternatives. A multicenter observational pilot validation study specifically focused on patients with BPH undergoing transurethral resection has also assessed the efficacy and reliability of app-based uroflowmetry for treatment monitoring [20]. These collective efforts underscore the growing recognition of the potential of acoustic uroflowmetry.

This study has several limitations. The generalizability of the findings may be initially limited to the specific population and smartphone models included in the validation study. Therefore, larger and more heterogeneous samples from a multicenter approach will eventually be necessary to confirm broader applicability. The restriction to male participants limits the applicability of the findings to female patients with LUTS. However, as detailed in the Methods section, a separate validation study among women may be performed in the future. Moreover, the potential for selection bias should also be acknowledged, as participants recruited from urology clinics may have more severe symptoms than the general population. The app does not aim to replace comprehensive urodynamic studies when indicated, but rather to provide a convenient screening and monitoring tool prior to formal urodynamic evaluation.

## Appendix

Multimedia Appendix 1 Peer review by the Indonesian National Research and Innovation Agency with Program Pendanaan Inovasi (PPI / Innovation Funding Program) 2026 grant scheme.

## Abbreviations

BPH: benign prostatic hyperplasia
CNN: convolutional neural network
FT: flow time
ICC: intraclass correlation coefficient
LOA: limits of agreement
LUTS: lower urinary tract symptoms
PCM: pulse-code modulation
Qavg: average flow rate
Qmax: maximum flow rate
RMS: root mean square
RNN: recurrent neural network
STARD-AI: Standards for Reporting Diagnostic Accuracy Studies for Artificial Intelligence
TQmax: time to maximum flow
TRIPOD: Transparent Reporting of a Multivariable Prediction Model for Individual Prognosis or Diagnosis
VV: voided volume

## Data Availability

The datasets generated and/or analyzed during the current study are available from the corresponding author on reasonable request.
