# Supplementary material for: Digital Acoustic Uroflowmetry for Noninvasive Urine Flow Rate Monitoring in Men Using Smartphone Acoustic Pattern Recognition: Protocol for the Development of a System and Mobile App
Source: JMIR Res Protoc. 2026 Jul 23;15:e102842. doi: 10.2196/102842 (PMC13448196; doi:10.2196/102842)
Supplement: Supplementary file 1 [file resprot_v15i1e102842_app1.pdf]

Reviewers' Comments for Author's Rebuttal

Manuscript titled "The **Digital Acoustic Uroflowmetry for Non-Invasive Urine Flow Rate Monitoring on Men using Smartphone Acoustic Pattern Recognition: Protocol for a System and Mobile App**"

Please write down your response or revision in **"Author's Response / Revision"** column. Locate the changes you've made or the sentence that you refer to as their page and line in **"Location in Text"** column. Please also mark the changes you have made **in your manuscript** by highlighting or changing the font color.

Reviewer

No.	Aspect	Comments	Author's Response / Revision	Location in Text
	Title	**REVIEWER 1**<br>-<br><br>**REVIEWER 2**<br>The use of acoustic should be added to the title as it is the primary data taken in the study	**REVIEWER 2**<br>The title has been changed accordingly.	Title
	Abstract	**REVIEWER 1**<br>-	**REVIEWER 2:**<br>1. The abstract was revised accordingly.	Abstract<br>Page 2

		**REVIEWER 2**<br>1. There are some grammatical errors and mistyping in the abstract		
	Introduction	**REVIEWER 1**<br>1. Data variability is a unique problem which should be anticipated in this study. The challenges of having standardized data despite having different smartphone specifications should be included<br>2. The availability of smartphone in developing countries such as Indonesia should be mentioned<br><br>**REVIEWER 2**<br>-	**REVIEWER 1:**<br>1. Added a section about the variable data and challenges on obtaining acoustics data from smartphones in this study<br>2. Added a section about data on ownership of smartphones in Indonesia	Introduction Page 3.
	Methods	**REVIEWER 1**<br>1. How to determine that the audio data taken from the smartphone are similar? Is there a calibration method included?<br>2. What about the patients who cannot use smartphones? Will there any special protocol for the population?<br><br>**REVIEWER 2**<br>1. The possible artificial intelligence used should be explained, is there any similar study using	**REVIEWER 1**<br>1. There will be calibration methods included in the application. Added to the methods section<br>2. Unfortunately, those who are unable to operate a smartphone application will be excluded from the study.<br><br>**REVIEWER 2**<br>1. The explanation about AI used in the study was added.	Methods

		proven infrastructure?	2. The picture will be taken by either the parent or the guardian, while the clinicians (pediatric urologists) would provide the label for machine training.	
	Results	**REVIEWER 1**<br>-<br><br>**REVIEWER 2**<br>-	-	Results
	Discussion	**REVIEWER 1**<br>1. Please cite previous studies with similar concept and infrastructure<br><br>**REVIEWER 2**<br>1. This study needs to anticipate not only the infrastructure limitation on the hardware (smartphones), but also the limitations on the user, such as bad signal or compliance issues	**REVIEWER 1**<br>1. Several studies are cited and explained in the discussion section.<br><br>**REVIEWER 2**<br>1. The limitations of the study were added	Discussion
